# Symptoms of eating disorders and low energy availability in recreational active female runners

**DOI:** 10.1136/bmjsem-2023-001623

**Published:** 2023-07-18

**Authors:** Elin Karlsson, Marie Alricsson, Anna Melin

**Affiliations:** Department of Sports Science, Linnaeus University, Kalmar, Sweden

**Keywords:** Endurance, Injury, Questionnaire, Sports and nutrition, Female athlete triad

## Abstract

**Objectives:**

This retrospective, cross-sectional study aimed to investigate symptoms of eating disorders (EDs) and low energy availability (LEA) among recreational female runners.

**Methods:**

Females (18–39 years) (n=89) participating in running group sessions organised by running clubs and companies were recruited via social media and completed an anonymous online survey compromising the Eating Disorder Examination Questionnaire (EDE-Q) and Low Energy Availability in Females Questionnaire (LEAF-Q). An EDE-Q global score ≥2.3 and a LEAF-Q total score ≥8 (in combination with an injury score≥2 and/or menstruation dysfunction score≥4) were used to categorise subjects as having symptoms of EDs and LEA, respectively.

**Results:**

Among the subjects fulfilling the age criteria (n=85), 18% (n=15) had symptoms of EDs and 19% (n=16) had symptoms of LEA. Of those with symptoms of EDs, 13% (n=2) had concomitant symptoms of LEA. The higher the EDE-Q dietary restraint score, the higher the gastrointestinal problem score (r=0.23, p=0.04), otherwise no other associations were found between EDE-Q global or subscale scores and LEAF-Q scores.

**Conclusion:**

Our results indicate that symptoms of EDs and LEA are frequent among adult females at all athletic levels, including the recreational level. Hence, to prevent the negative health consequences of EDs and LEA, preventative initiatives are also needed in recreational running communities.

**What is already known on this topic**

- Early detection of athletes at risk of eating disorders (EDs) and low energy availability (LEA) is important to prevent adverse long-term health effects and impaired sports performance.
- Female athletes in leanness sports such as long-distance running have an increased risk of EDs and LEA.
- Most athletes with LEA have a body mass within the normal range, while athletes with EDs may have a body mass below, equal to or above the normal reference range.

**What this study adds**

- Although not concomitant, symptoms of EDs and LEA are frequent among adult recreational female runners.
- Just like athletes, most recreational female runners with and without symptoms of EDs or LEA have a body mass within the normal reference range.

**How this study might affect research, practice or policy**

- To prevent negative health consequences of EDs and severe LEA, there is a need for preventative initiatives not only among elite athletes but also among recreational female runners and in their running communities.

## Introduction

Eating disorders (EDs) are serious mental illnesses with high mortality rates.^1^ The development of behaviours associated with EDs may be described as a continuum that starts with appropriate eating and exercise behaviours, that may progress to chronic dieting and severe low energy availability (LEA), and the continuum ends with clinical EDs.^2^ The underlying causes of LEA are besides EDs manifold and include well-planned and supervised weight-regulation periods as well as inadvertent undereating such as poor appetite or lack of sports nutritional knowledge.^3^ Severe LEA with or without EDs may lead to impaired health (eg, menstrual dysfunction, bone stress injuries and gastrointestinal problems)^4^ and sports performance (eg, reduced strength and endurance, reduced training response and increased risk of injuries).^5^ Therefore, early detection of athletes at risk for EDs and severe LEA is important to prevent adverse long-term health effects.^4^ A few validated screening tools are available to assist in the identification of symptoms of EDs and LEA, such as restrictive eating behaviour,^2^ and menstrual dysfunction in female athletes.^6^

The reported prevalence of EDs is higher in female athletes, especially in leanness sports such as long-distance running compared with athletes in non-leanness sports and recreationally active females.^7^ Furthermore, athletes in leanness sports may be at particular risk of LEA due to sport-specific demands of a low body mass^2^ and/or challenges to consume an adequate amount of energy to match high exercise energy expenditures.^5^ However, the prevalence of symptoms of EDs and LEA among recreationally active people has been sparsely investigated. Therefore, the present study aimed to investigate symptoms of EDs and LEA among recreational active female runners.

## Methods

### Study design and patient involvement

In this retrospective cross-sectional study, recreational female runners (18–39 years) participating in running group sessions organised by running clubs and companies via a total of 37 Facebook groups were invited to complete an anonymous online survey. After obtaining permission from the clubs and companies, detailed information about the procedure and purpose of the study and a link to the online survey were shared in the Facebook groups. Informed consent was obtained by filling out the survey. The online survey was created by using Google Forms compromising two validated screening instruments: the Eating Disorder Examination Questionnaire (EDE-Q)^8^ and the Low Energy Availability in Females Questionnaire (LEAF-Q),^6^ including information regarding age, height, body mass, weight fluctuation (highest vs lowest body mass with current height) and average training hours per week. The subjects or the public were not involved in the design, conduct, reporting or dissemination plans of the research.

### Eating Disorder Examination Questionnaire

Symptoms of EDs were evaluated using the EDE-Q,^8^ which is considered a useful instrument to identify both recreational and elite athletes with ED symptoms.^9^ In this study, V.6.0 of EDE-Q was used, which consists of 28 items divided into four subscales: (1) restraint (restriction and avoidance of eating), (2) eating concern (preoccupation with thoughts and anxiety about food and eating), (3) shape concern (body dissatisfaction and discomfort) and (4) weight concern (preoccupation with body mass and desire to lose weight).^8^ Hence, the EDE-Q measures cognitive and behavioural symptoms related to EDs, focusing on the last 28 days, and rated using a Likert scale (0–6) where higher scores reflect greater ED psychopathology.^8^ The item scores within each subscale were summed up and averaged to provide subscale scores, and an EDE-Q global score was calculated by summering and averaging the four subscale scores. An EDE-Q global score of ≥2.3 was used to categorise subjects as having symptoms of EDs. This cut-off score has been reported to be the optimal cut-off score in a sample of 195 adults with a specificity of 86% and a sensitivity of 92%.^10^

### Low Energy Availability in Females Questionnaire

The LEAF-Q was used to identify subjects with symptoms of LEA.^6^ The questionnaire consists of 25 items divided into three variables: (1) menstrual dysfunction (including questions regarding hormonal contraceptive use), (2) sports injuries and (3) gastrointestinal problems rated using a Likert scale. The LEAF-Q was first validated among elite female endurance athletes where a LEAF-Q total score ≥8 provided a sensitivity of 78% and a specificity of 90% for correctly classifying current LEA and/or low bone mineral density and/or menstrual dysfunction.^6^ More recently, LEAF-Q was validated in a mixed-sport cohort of elite and pre-elite female athletes where the injury and menstrual dysfunction cut-off scores proved a sensitivity of 100% and 80%, respectively, for the detection of low bone mineral density and self-reported menstrual dysfunction.^11^ Considering the results from the validation studies by Melin *et al*^6^ and Rogers *et al*,^11^ the subjects in the present study were categorised as having symptoms of LEA as a LEAF-Q total score ≥8 in combination with an injury score ≥2 (subjects with or without hormonal contraceptive use) and/or menstruation dysfunction score ≥4 (subjects without hormonal contraceptive use). Current menstrual status was determined among subjects not using a hormonal contraceptive since hormonal contraceptives ensure cyclic bleeding due to exogenous oestrogen independently of endogenous hormonal status.^12^ Subjects were categorised as having menstrual dysfunction if they reported either <9 menstrual cycles/year and/or the absence of >3 consequent menstrual cycles. Previous menstrual dysfunction was defined as primary amenorrhoea (menarche at an age of ≥15 years) or secondary amenorrhoea (the absence of >3 consequent menstrual cycles).^13^ Days absent or marked limitation from participation in training or competition due to sports injuries in the previous year were evaluated. A severe sports injury was defined as a time loss from training or competition ≥22 days.^14 15^ Subjects were categorised as having gastrointestinal problems if they reported either (1) feeling gaseous or bloated in the abdomen almost every day and/or several times a week and/or (2) having cramps or stomachache several times a day and/or several times a week and/or (3) having bowel movements on average once a week or more rarely and/or twice a week and/or (4) having stool consistency as diarrhoea-like (watery) and/or hard and dry.^6^

### Statistical analysis

Data from the Google Forms survey were transferred into and compiled in Microsoft Excel V.12.43. Statistical analyses were performed using IBM SPSS Statistics for Macintosh V.27. The Shapiro-Wilk test was used to test for normality in the continuous variables. Normally distributed continuous data were expressed by mean±SD and non-normally distributed data as median and IQR (25 and 75 percentiles). For categorical data, results were expressed as numbers (n) and percentages (%). To investigate differences between groups independent t*-*test was used for normally distributed continuous data and Mann-Whitney U test was used for non-normally distributed continuous data. The chi-square test was used to test for differences between categorical data. Spearman’s correlation coefficient (r) was calculated to measure the degree of a positive or negative association between continuous variables. The significance level was set at <0.05 for all analyses.

## Results

In total, 89 subjects completed the online survey. Four were excluded due to age >39 years resulting in 85 subjects being included in the final sample and the analysis. Descriptive characteristics are presented in table 1. Two subjects (2%) were underweight (body mass index (BMI) <18.5 kg/m^2^), while 11 (13%) were overweight (BMI≥25 kg/m^2^) and 1 (1%) was obese (BMI≥30 kg/m^2^).^16^ The subjects’ habitual average training load was 5 hours per week, ranging from 1 to 18, of which 80 subjects (94%) were classified as recreationally active, four (5%) as sedentary, and one (1%) as trained/developmental athlete. The majority of the subjects were, therefore, classified as tier 1 athletes.^17^

**Table 1:** The descriptive characteristics of the total sample and the results of EDE-Q divided by subjects with vs no symptoms of EDs

	All (n=85)	Symptoms of EDs (n=15)	No symptoms of EDs (n=70)	P value
Age (years)	32.4±4.3	30.1±4.5	32.9±4.1	0.023
Height (cm)	167.5±5.9	167.0±3.4	167.6±6.3	0.742
Weight (kg)	62.8±8.4	65.1±12.0	62.3±7.4	0.243
Weight fluctuation (kg)	11.0 (7.5–15.5)	13.0 (10.5–16.0)	10.0 (7.0–15.5)	0.089
BMI (kg/m^2^)	22.0 (20.7–23.7)	22.3 (20.5–25.0)	21.7 (20.8–23.4)	0.454
Exercise (hours/week)	5.0 (3.5–6.0)	5.0 (3.0–6.0)	5.0 (3.5–6.1)	0.917
Global score	1.2 (0.5–1.8)	3.3 (2.7–3.7)	0.9 (0.5–1.4)	<0.001
Dietary restraint	0.4 (0.0–1.3)	3.0 (2.4–3.8)	0.0 (0.0–0.5)	<0.001
Eating concern	0.4 (0.2–0.9)	1.8 (0.8–2.6)	0.2 (0.2–0.6)	<0.001
Shape concern	1.6 (0.9–3.0)	4.3 (3.8–4.9)	1.4 (0.8–2.3)	<0.001
Weight concern	1.4 (0.8–2.4)	3.6 (3.4–4.2)	1.1 (0.6–1.8)	<0.001

Data are presented as mean±SD for normally distributed data and as median and IQR (25–75) for non-normally distributed data.

Significance levels were set to p <0.05.

Weight fluctuation refers to the highest vs lowest body mass with the current height.

BMI, body mass index; EDE-Q, Eating Disorder Examination Questionnaire; EDs, eating disorders.

### Symptoms of EDs

Fifteen subjects (18%) had symptoms of EDs, and besides being younger no differences were found in body mass, BMI or training hours per week compared with subjects without symptoms of EDs (table 1). The shape concern score was the highest of all EDE-Q subscale scores, followed by weight concern, whereas the subscales restraint and eating concern presented the lowest scores. The EDE-Q global score was positively associated with weight fluctuation (r=0.22, p=0.045), the restraint score with BMI (r=0.22, p=0.044), and eating concern score with age as well as weight fluctuation (r=0.22, p=0.046; r=0.29, p=0.008, respectively).

### Symptoms of LEA

Sixteen subjects (19%) had symptoms of LEA (table 2) of which 2 (13%) also had symptoms of EDs. There were no differences in any LEAF-Q variables between subjects with and without symptoms of EDs.

**Table 2:** Results from the LEAF-Q for the total sample and divided by subjects with vs no symptoms of EDs

	All (n=85)	Symptoms of EDs (n=15)	No symptoms of EDs (n=70)	P value
Total LEAF-Q score	4.0 (2.0–6.0)	5.0 (3.0–6.5)	5.5 (2.0–8.0)	0.378
Subjects with symptoms of LEA, % (n)	18.9 (16)	13.3 (2)	20.0 (14)	0.549
Gastrointestinal problem score	2.0 (0.5–3.0)	3.0 (0.0–4.0)	2.0 (1.0–4.0)	0.153
Subjects with gastrointestinal problems, % (n)	24.7 (21)	33.3 (5)	22.9 (16)	0.393
Injury score	1.0 (0.0–3.0)	1.0 (0.0–3.0)	2.0 (0.0–5.0)	0.921
Subjects with severe sports injury % (n)	15.3 (13)	6.7 (1)	17.1 (12)	0.262
Menstrual dysfunction score	1.0 (0.0–2.0)	0.0 (0.0–1.5)	1.0 (0.0–2.0)	0.601
Subjects with menstrual dysfunction, % (n), (n=41)	14.6 (6)	12.5 (1)	15.2 (5)	0.849

Data are presented as median and IQR (25–75) and the number of subjects is shown as number (n) and percentage (%).

Significance levels were set to <0.05.

EDs, eating disorders; LEA, low energy availability; LEAF-Q, Low Energy Availability in Females Questionnaire.

Subjects with symptoms of LEA trained more compared with those without symptoms (7.0 hours per week (4.5–8.0 hours per week) vs 4.5 hours per week (3.0–6.0 hours per week), p<0.001)); otherwise, no differences were found in age, body mass or weight fluctuation between subjects with and without symptoms of LEA. LEAF-Q total score was positively associated with average training hours per week (r=0.33, p=0.002) and negatively associated with age (r=−0.37, p<0.001).

Of the 6 subjects with menstrual dysfunction, 4 reported <9 menstrual cycles per year and 2 reported absence of >3 consequent menstrual cycles. Five subjects reported using oral contraceptives to prevent cessation of menstruation and/or had experienced cessation of menstruation with increased exercise load. Of the total sample, previous menstrual dysfunction was reported by 24 subjects (28%) where 14 (16%) were defined as primary amenorrhoea and 10 (12%) as secondary amenorrhoea.

Almost half of the subjects (48%, n=41) reported being absent or markedly limited from participation in training or competition due to a sports injury in the previous year, and 15% reported a severe sports injury. Subjects who reported having had a sports injury were younger (31.1±4.5 years vs 33.5±3.9 years, p=0.016) and trained more (5.5 hours per week (4.0–7.0 hours per week) vs 4.0 hours per week (3.0–6.0 hours per week), p=0.024) compared with those who did not report any sports injury.

Twenty-one percent of the subjects reported gastrointestinal problems, and the EDE restraint score was positively associated with the gastrointestinal problem score (r=0.228; p=0.036); otherwise, no other associations were found between EDE-Q global or subscale scores and LEAF-Q scores. No associations were found between menstrual dysfunction, injury and gastrointestinal problem scores.

## Discussion

The present study aimed to investigate symptoms of EDs and LEA among recreational female runners. The main finding was that 18% of the females had symptoms of EDs and 19% had symptoms of LEA, while the coexistence of symptoms of EDs and LEA rarely occurred with only two subjects having symptoms of both.

### Symptoms of EDs

In the present study, 18% of these recreational runners had symptoms of EDs, similar to the 18%–21% reported among female endurance athletes.^18 19^ In studies using clinical diagnostic interviews to confirm symptoms of EDs, the reported prevalence of EDs has been 24%–25% among female elite endurance athletes,^20 21^ 21% among recreationally active females^7^ compared with 9% in the general population.^7 21^ In a meta-analysis, Chapa *et al*^22^ found that ED psychopathology varies in females and non-athletes depending on sport type, rather than on the competitive level with a higher ED psychopathology in aesthetic or leanness sports. In summary, the results from the present study demonstrate a concerningly high prevalence of symptoms of EDs in recreationally active females, comparable to that observed among the athletic population.

Depending on the ED symptoms and severity, athletes with EDs may be underweight, normal weight or overweight.^2^ In the present study, no difference in body mass was found between the subjects with and without symptoms of EDs which is in line with a study by Torstveit *et al*^7^ reporting an average body mass within the normal range in female elite athletes with clinical EDs. Also among females with or without symptoms of LEA, similar body masses have been reported,^20 23–25^ supported by the findings in the present study. These findings suggest that most females with symptoms of EDs or LEA often have a body mass within the normal reference range.^3 26^ Hence, body mass is not a useful indicator for identifying athletes or recreational active females with symptoms of EDs or LEA. Weight fluctuations are commonly reported among young female athletes with disordered eating behaviour or EDs,^27^ supported by the findings in this study where weight fluctuation was positively associated with EDE-Q global and eating concern scores. Moreover, restraint score was positively associated with higher BMI. These findings might reflect a preoccupation with thoughts and anxiety about food and eating and an intent to limit food intake^8^ whether or not the effort is successful.

The shape and weight concern subscales represented the highest scores, aligning with findings from previous studies among female athletes.^18 19 28^ In fact, non-athletes report more body dissatisfaction compared with athletes.^22^ With this in mind, it might be important to emphasise a positive body image, physical and psychological health benefits, and healthy dietary habits among recreational female runners.

### Symptoms of EDs and LEA

In the present study, only 13% of the subjects with EDs had symptoms of LEA, and no associations were found between EDE-Q global and LEAF-Q scores. This finding confirms the broad spectrum of symptoms associated with EDs and highlights that EDs can manifest in various ways, including behaviours that may not necessarily result in undereating but still compromise the individual’s physical or mental health.^29^ Conversely, the findings in the present study emphasise that recreational female runners may experience symptoms of LEA in the absence of EDs symptoms. This is confirmed by previous research where 27% of female endurance athletes^19^ and 20% of female adolescent athletes^30^ had symptoms of both EDs and LEA. The relatively low prevalence of concurrent symptoms of EDs and LEA in this study, compared with previous research^19 30^ could potentially be attributed to the recreational activity level of the study subjects. Moreover, the finding in the present study that a higher weekly training load was associated with symptoms of LEA, raises the speculation that the subjects might not be aware of the energy cost of their exercise or are unable to consume an adequate amount of energy to match it.

In this study, 19% of the subjects had symptoms of LEA, similar to the 23% reported among Irish recreational active females^24^ but lower compared with the 45% and 63% reported in recreationally active females in New Zealand.^25 31^ The mean age of the subjects in the present study was 32 years and both symptoms of EDs and a higher LEAF-Q total score were associated with lower age, in contrast to previous research where a similar prevalence of ED psychopathology across age groups has been reported.^22^ Moreover, clinical studies have found that females with a gynaecological age <14 years are more sensitive to LEA than older females.^32^ It is, therefore, possible that the lower mean age (23–24 years) in the studies of recreational active females in New Zealand^25 31^ potentially could explain the higher reported prevalence of LEA compared with the present study. Consequently, the relatively low prevalence (15%) of menstrual dysfunction in the present study may be attributed to the subjects’ higher age and potentially reduced sensitivity to LEA.

In the present study, no associations were found between sports injuries and symptoms of EDs or menstrual dysfunction, which is in contrast to earlier studies reporting more sports injuries among young female athletes with EDs symptoms^15^ and menstrual dysfunction.^14 15 33^ However, the present study cannot conclusively determine if the reported sports injuries, menstrual dysfunction and gastrointestinal problems, as indicated by the LEAF-Q responses, are specifically caused by LEA, or could be attributed to acute injuries or other conditions compromising reproductive and gastrointestinal functions.

### Strengths and limitations of the study

In this study, we combined two validated screening tools to better understand the associations between symptoms of EDs and LEA.^34^ Although LEAF-Q has been frequently used for screening for symptoms of LEA in populations with various athletic levels, including recreational active females, its validation has been limited to athletes.^6 11^ Due to the study’s cross-sectional design, it is not possible to determine the cause and effect of the results and the results are, therefore, limited to prevalence estimations. Furthermore, the study may be susceptible to ascertainment bias, specifically in terms of selection bias. It is possible that older females are more likely to participate in a survey compared with younger females, which could have resulted in a relatively high mean age (32 years) among the subjects. This potential bias as well as the convenience sample could limit the generalisability of the findings to the broader population of recreational female runners. Moreover, retrospective self-reported data in this study may be subject to recall bias and reporting bias, depending on the subject’s interpretation of the questions, honesty of completion and ability to accurately recall the data. For instance, under-reporting of eating pathology^7^ and difficulties remembering details of injury history such as the number of days injured is common.^35^ However, recalling injury status in the previous year (injured/uninjured) is considered accurate^35^ and self-reported menstrual dysfunction is a valid predictor for clinical menstrual dysfunction among athletes.^6^

Screening for symptoms of EDs and LEA may be a convenient method for identifying individuals who need to be encouraged to seek medical attention.^3^ To accurately confirm and differentiate the conditions, future studies among recreational females may, however, wish to investigate EDs and LEA using clinical interviews, biomarkers (eg, sex and thyroid hormones) and examinations (eg, bone mineral density and ovulation).

## Conclusions

This study indicates that symptoms of EDs and LEA are frequent among adult recreational female runners—a population where previous research in this area has been sparse. Hence, to prevent the negative health consequences of EDs and severe LEA, preventative initiatives are also needed in recreational running communities. More studies are needed to investigate EDs and clinical outcomes related to LEA among recreationally active people.

## Data Availability

Data sets are available from the corresponding author upon reasonable request.
